# Patterns and trends of medicinal poisoning substances: a population-based cohort study of injuries in 0–11 year old children from 1998–2018

**DOI:** 10.1186/s13690-024-01268-7

**Published:** 2024-04-16

**Authors:** Edward G Tyrrell, Elizabeth Orton, Laila J Tata, Denise Kendrick

**Affiliations:** 1 Centre for Academic Primary Care, Lifespan and Population Health, School of Medicine, University of Nottingham, Nottingham, UK; 2 Lifespan and Population Health, School of Medicine, University of Nottingham, Nottingham, UK

**Keywords:** Injury, Accident, Child safety, Child public health, Epidemiology, Social deprivation

## Abstract

**Background:**

There have been sharp increases in antidepressant and opioid prescriptions over the last 10 years, as well as increased over-the-counter medicine availability. However, the impact on childhood medicinal poisonings rates, particularly by socioeconomic deprivation is unclear. This study reports population level medicinal poisoning substance patterns in England among children aged 0–11 years, helping inform safety advice and poisoning prevention interventions.

**Methods:**

An open cohort study of 1,489,620 0–11 year olds was conducted from 1998 to 2018, using the Clinical Practice Research Datalink, to examine inpatient hospital admissions for poisoning. Incidence rates and adjusted incidence rate ratios (aIRR) were calculated for poisoning substance groups by age, sex, socio-economic deprivation and year.

**Results:**

3,685 medicinal poisoning hospital admissions were identified. The most common substances were paracetamol (33.2%), dependence/withdrawal risk drugs (DWRD - antidepressants, opioids, gabapentinoids, benzodiazepines) (13.5%) and other over-the-counter (OTC) analgesics/anti-common cold drugs (13.0%). Over the study period DWRD poisonings decreased 33% (aIRR 0.67, 95%CI 0.50–0.90 comparing 2013/14-2017/18 to 1998/99-2002/03), while paracetamol poisonings increased 43% (aIRR 1.43, 95%CI 1.20–1.70 for the same periods), with no change in incidence rates for other OTC drugs (aIRR 0.82, 95% CI 0.60–1.12) or all medications combined (aIRR 0.97, 95%CI 0.88–1.07). A gradient in poisonings by area-level socioeconomic deprivation was shown for all medications (aIRR 1.32, 95%CI 1.18–1.47 for most deprived compared to least deprived quintile), and DWRDs (aIRR 2.03, 95%CI 1.42–2.88 for 4th most deprived quintile and aIRR 1.88, 95%CI 1.32–2.66 for 5th most deprived quintile, compared to least deprived quintile), but not for paracetamol or other OTC drug poisonings.

**Conclusions:**

Poisonings from DWRDs decreased by 33%, while paracetamol poisonings increased by 43% during the study period. There was a gradient by area-level socioeconomic deprivation in prescribed medication poisonings, including drugs with withdrawal/dependence risk, but not OTC medication poisonings. Households in more socioeconomically deprived areas have the potential to benefit most from measures to improve safe storage of medicines and are likely to require targeted interventions providing education and safety equipment. In addition, universal promotion of the safe storage of OTC and prescribed medicines must be provided by prescribers, community pharmacies and other outlets of such medication.

**Supplementary Information:**

The online version contains supplementary material available at 10.1186/s13690-024-01268-7.

**Table:** 

Text box 1. Contributions to the literature
• UK population level childhood poisoning substance data is lacking, but needed to inform medication safety advice and policies
• From 1998 to 2018 childhood poisoning hospital admissions from common prescribed medications decreased 33%, but from paracetamol increased 43%.
• Children in highly socio-economically deprived areas were twice as likely to have a prescribed medication poisoning, but no more likely an over-the-counter medication poisoning, as children in least deprived areas.
• Households in more deprived areas have greatest potential to benefit from measures to improve prescribed medicine safe storage, likely requiring targeted interventions.
• Safe over-the-counter medication storage must also be universally promoted by medication outlets.

## Introduction

Childhood poisonings are an important cause of preventable ill health and cause significant distress to both children and their families. Although many poisonings are preventable, in England they result in an estimated 26,000 emergency hospital attendances annually among 0–4 year olds [1] and roughly 4,500 hospital admissions in 0–9 year olds [2]. The English Chief Medical Officer’s report from 2012 made a strong economic case for preventing childhood injuries [3]. 

Over the last 50 years in the UK there has been significant progress in reducing poisonings, in particular poisoning deaths among children [4]. Much of this has been credited to the success of public health interventions, such as introducing child-resistant packaging [5]. However, more recent increased availability of over-the-counter medicines alongside increases in prescribed medicine in the UK over the last 15 years [6], particularly sharp increases in prescriptions of drugs like antidepressants [7] and opioids [8], may have influenced poisoning incidence, and this has not been previously investigated.

Although some previous studies have reported the incidence and socio-demographic risk factors for poisoning in children under the age of 12 in the UK, these are now out of date [9–13] and often used data from single hospitals [11–14]. Three studies examined poisonings at a population level up to 2011 at the latest [15–17], but did not examine the specific medicinal substances involved. Mbeledogu et al. reported a 23% reduction in hospital admission rates from poisoning in 0-4-year-olds between 2000 and 2011, with an upward trend over the last 2 years studied [16], with most (77%) poisoning admissions involving medicines. Despite a reduction in the deprivation gradient in poisonings over time, in 2011 those living in the most deprived areas still had a 54% higher incidence of poisoning admission than those in the least deprived areas. The other two studies examined epidemiological patterns and trends in poisonings as part of wider studies of incidence across different types of injury [15, 17]. 

Our study aim was to estimate population level incidence of poisonings leading to hospital admission among 0–11 year olds in England between 1998 and 2018 according to the most common poisoning substances, sex, age, deprivation level, geographical area and changes over time. We therefore, report the most complete and detailed population level estimates of medicinal poisoning for the most common substances in children and trends to date in England. This will help inform safety advice offered to families as well as the commissioning and delivery of poisoning prevention interventions.

## Methods

### Study population

This study utilised routinely collected general practice data from the Clinical Practice Research Datalink (CPRD) including information on hospital admissions, from linked Hospital Episode Statistics (HES) data (which records all hospital admissions in England). In the UK, 98% of the population is registered with a general practitioner (GP) [18]. 

The study population was an open cohort capturing the registered patient population in CPRD Gold with HES data available, aged 0–11 years old between April 1st 1998 and March 31st 2018. The cohort were from 399 general practices across England. Children entered the cohort on the latest of their date of birth, registration date with their general practice, date the practice reached the CPRD defined data quality standard for completeness, or first date of data collection from the practice within the study period. They exited the cohort at the earliest of their 12th birthday, date of death, date they left the practice, or last date of data collection from the practice within the study period.

### Outcome events

The study outcome was an inpatient hospital admission from a poisoning event involving at least one medicinal substance, within the study period. Repeat poisoning events within the same individual were included.

Poisonings, and the substances involved, were identified using International Classification of Diseases, 10th revision (ICD-10) codes (T36-50 and X40-44) [19] in HES data. A single admission can have multiple codes and the poisoning codes could occur at any position within the HES data. Poisoning substances were categorised using ICD-10 categories T36-50 and grouped into three groups representing the most common poisoning substances: paracetamol, drugs with a risk of dependence/withdrawal and other over the counter medications (see below for ICD-10 codes included in each group). All substances recorded for a poisoning event have been included. Therefore, one poisoning event may have had more than one contributing substance.

Outcome events were limited to hospital admissions from poisoning for two reasons. The National Institute for Health and Care Excellence (NICE), who provide clinical guidelines in the UK, suggest that admission should be considered for all instances of childhood poisoning [20], making it comparatively unlikely that relevant poisoning events will be managed without admission. Furthermore, poisonings recorded only in primary care data have been previously shown to lack detail of the specific poisoning substances involved [9], limiting the utility of these data for this study.

### Statistical analysis

The number and percentage of poisoning events involving specific substances was identified. The most commonly occurring substances were combined into groups based on ICD-10 category and clinical relevance. The 3 groups were:

- Paracetamol (T39.1 (4-Aminophenol derivatives)) – included individually based on its common involvement in poisoning events.
- Dependence/withdrawal risk drugs (DWRD) – includes opioids (T40.2 (Other opioids), T40.3 (Methadone), T40.4 (Other synthetic narcotics)), benzodiazepines (T42.4), gabapentinoids (T42.6 (Other anti-epileptic and sedative hypnotic drugs)) and antidepressants (T43.0 (Tricyclic and tetracyclic antidepressants), T43.1 (Monoamine-oxidase-inhibitor antidepressants), T43.2 (Other and unspecified antidepressants (*includes SSRIs*)). This grouping was based on a Public Health England report outlining increases in the prescription of such medications over the previous 10 years and their potential dangers [21]. We therefore concluded this was an appropriate group of medications to study together from a clinically relevant perspective.
- Other over–the-counter (OTC, meaning they can be bought without the need for a prescription) analgesics and anti-common cold drugs (subsequently referred to as Other OTCs) – includes ICD-10 categories T39.0 (Salicylates (*aspirin*)), T39.3 (Other nonsteroidal anti-inflammatory drugs [NSAID]) and T48.5 (Anti-common-cold drugs).

Incidence rates (IR) of hospital admission for poisoning per 100,000 person-years (PY) and 95% confidence intervals (95%CI) were calculated for the most common substance groups, as well as for all medicinal poisonings combined. They were estimated overall and separately for boys and girls, by age, socio-economic deprivation quintiles, and calendar year. Adjustment was also made for geographical region.

Age and year were included as time-varying covariates by Lexis expansion. This is a method of accounting for the changing age of individual participants or of time-period, as time elapses through the study. It involves calculating age specific or time-period specific rates, allowing each cohort member to contribute data in more than one age group or time period. Time-period was analysed in single years to calculate incidence rates then in 5-year bands to calculate incidence rate ratios (IRR). Socio-economic deprivation was assessed using quintiles of the Index of Multiple Deprivation 2010 (IMD) for England [22]. This was provided by CPRD, at a lower super output area level (approximately 650 households), based on the postcode of the individual’s GP practice.

Unadjusted incidence rate ratios (aIRR) were calculated for all medicinal poisonings combined and for the most common substance groupings (paracetamol; DWRD; Other OTCs) using negative binomial regression. In addition, IRRs were adjusted (aIRR) for sex, age, calendar period, socio-economic deprivation and geographical region, which were identified *a priori* as important relevant confounders of each other’s association with childhood poisoning events. The IRRs give a ratio of the incidence rates (of hospital admission for poisoning per 100,000 person-years) across different categories for each variable. Person-time was included as an offset in all models. Potential interactions were assessed including sex and age [23, 24], time-period and sex [24], time-period and age [25], time-period and socio-economic deprivation, with *p* < 0.01 taken as significant due to the large size of the dataset. Data were analysed using StataSE v16.

## Results

The cohort contained 1,489,620 children contributing 6,530,174 person-years (PY) of follow-up. Male children represented 52% of those without a poisoning and 53% of those with a poisoning (Table 1). Children with a poisoning entered the cohort at a younger age (median 0.2 years, inter-quartile range (IQR) 0.1–1.6) and contributed more person-time (median 6.5 PY, IQR 3.6–10.0) than those without a poisoning (median age entering cohort 2.1 years, IQR 0.1–6.6, median PY contributed 3.4, IQR 1.4–6.8). Children from areas with higher socioeconomic deprivation were more likely to experience a poisoning.

**Fig. 1:**
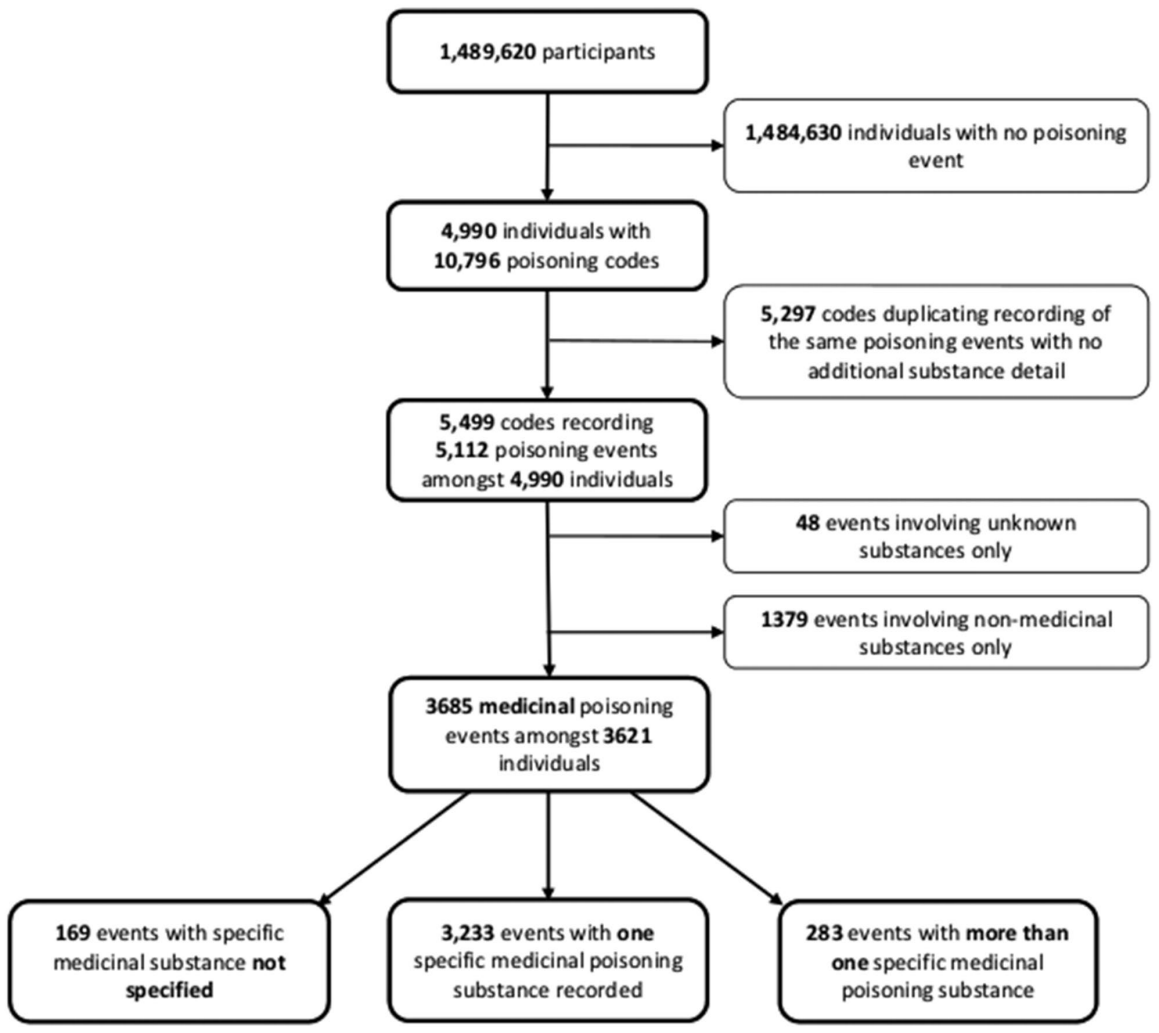
Flowchart identifying number of poisoning codes and events analysed

In total, 3,621 children experienced 3,685 medicinal poisoning events resulting in hospital admission (Fig. 1). Of all poisoning events, 3,516 (95.4%) had the specific poisoning substance(s) recorded. 3,233 events (87.7%) had a single medicinal substance recorded and 283 (7.7%) had more than one poisoning substance recorded.

**Table 1:** Characteristics of the cohort (*N* = 1,489,620 children age 0–11 between 1998–2018)

Characteristics	Number (%) of children (*N* = 1,489,620)	
	Children with no medicinal poisoning event (*N* = 1,485,999)	Children with at least one medicinal poisoning event (*N* = 3621)
Sex		
Male	766,607(52)	1923 (53)
Female	719,392(48)	1698 (47)
Age (median years, IQR*) entering cohort	2.1 (0.1–6.6)	0.2 (0.1–1.6)
Age (median years, IQR*) at first substance specific poisoning event		2.6 (1.9–3.5)
Person-years (median, IQR*) contributed	3.4 (1.4–6.8)	6.5 (3.6–10.0)
Socio-economic deprivation quintile†		
1 (least deprived)	236,244 (16)	472 (13)
2	273,987 (19)	617 (17)
3	282,356 (19)	615 (17)
4	316,229 (21)	768 (21)
5 (most deprived)	377,183 (25)	1149 (32)

*IQR – inter-quartile range

†Based on index of multiple deprivation (IMD) quintile

### Most common substances

When grouped by category, paracetamol was the most common poisoning substance (33.2%, Table 2), followed by DWRD (13.5%) and Other OTCs (13.0%). The number of poisoning events by specific ICD-10 substance category is in supplementary Table 1.

**Table 2:** Poisoning episodes by medicinal substance group (T36-T50)

Subgroup of medicinal poisonings (T36-50):	Description/codes included	Number of poisoning events involving substance *N* = 3,685	% of events involving substance (% of 3,685)
Paracetamol	T39.1 (4-Aminophenol derivatives *(paracetamol))*	1225	33.2
Dependence/ withdrawal risk drugs	T40.2 (Other opioids), T40.3 (Methadone), T40.4 (Other synthetic narcotics), T42.4 (Benzodiazepines), T42.6 (Other anti-epileptic and sedative hypnotic drugs), T43.0 (Tricyclic and tetracyclic antidepressants), T43.1 (Monoamine-oxidase-inhibitor antidepressants), T43.2 (Other and unspecified antidepressants *(includes SSRIs)*)	498	13.5
Other OTC* analgesics/anti-common cold drugs	T39.0 (Salicylates *(aspirin)*), T39.3 (Other nonsteroidal anti-inflammatory drugs [NSAID]), T48.5 (Anti-common-cold drugs)	482	13.0

*OTC – over-the-counter

### Age and sex variations

**Fig. 2:**
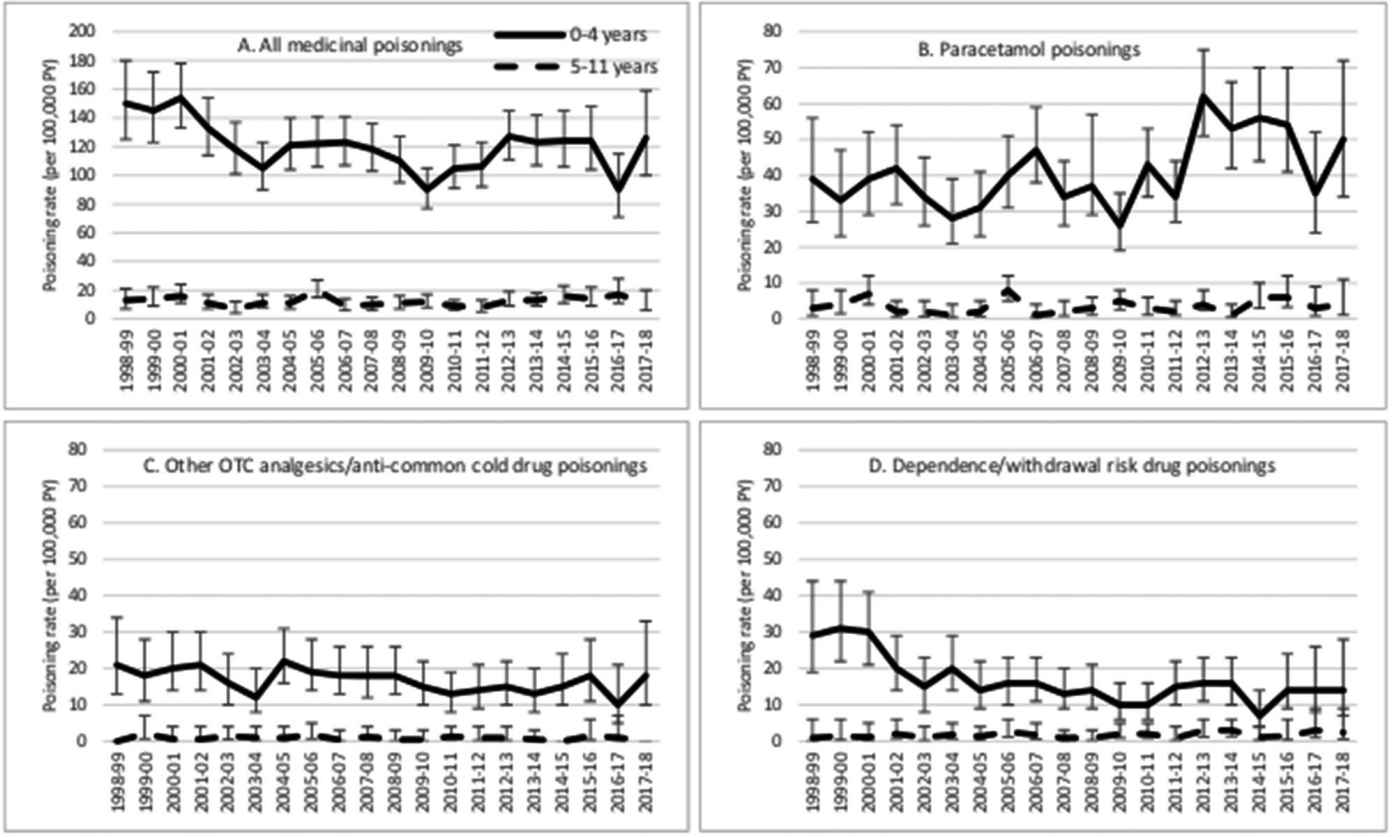
Incidence rates by age group over time, including 95% confidence intervals for **A** All medicinal poisonings, **B** Paracetamol poisonings, **C** Other over-the-counter analgesics/anti-common cold drug poisonings and **D** Dependence/withdrawal risk drug poisoning

There was no evidence of any interaction between the variables assessed. Poisoning incidence was higher for all poisoning substances in children aged 0–4 years than in children aged 5–11 years (Fig. 2). After adjusting for other factors, there was a 90% reduced risk of any medicinal poisoning for children aged 5–11 years compared with children aged 0–4 years (adjusted incidence rate ratio (aIRR) 0.10, 95% confidence interval (95%CI) 0.09–0.11), with similarly lower poisoning risk for older children shown for each of the most common substance groups (Table 3). After adjusting for other factors, there was a 7% lower risk of poisoning for females (aIRR 0.93, 95%CI 0.87–0.99) than males for all medicinal poisonings combined, but there was no difference in risk between male and female children for any of the specific substance groups examined.

**Table 3:** Unadjusted and adjusted poisoning incidence rate ratios

	Poisoning incidence rate ratios (95% confidence intervals)							
	All medicinal poisonings combined		Paracetamol (T39.1)		Other OTC analgesia/anti-common cold drugs (T39.0, T39.3, T48.5‡)		Dependence/withdrawal risk drugs (T40.2, T40.3, T40.4, T42.4, T42.6, T43.0, T43.1, T43.2‡)	
	**Unadjusted IRRs**	Adjusted IRRs*	**Unadjusted IRRs**	Adjusted IRRs*	**Unadjusted IRRs**	Adjusted IRRs*	**Unadjusted IRRs**	Adjusted IRRs*
Sex								
Male	1.00	1.00	1.00	1.00	1.00	1.00	1.00	1.00
Female	0.93 (0.86–1.00)	0.93 (0.87–0.99)	0.92 (0.82–1.03)	0.98 (0.83–1.16)	1.18 (0.95–1.45)	1.16 (0.96–1.40)	0.97 (0.78–1.20)	0.96 (0.79–1.15)
Age group (years)								
0–4	1.00	1.00	1.00	1.00	1.00	1.00	1.00	1.00
5–11	0.10 (0.09–0.11)	0.10 (0.09–0.11)	0.08 (0.07–0.10)	0.08 (0.07–0.10)	0.05 (0.03–0.07)	0.05 (0.03–0.07)	0.10 (0.07–0.13)	0.10 (0.07–0.13)
Calendar period								
1998/99-2002/03	1.00	1.00	1.00	1.00	1.00	1.00	1.00	1.00
2003/04-2007/08	0.87 (0.79–0.96)	0.89 (0.82–0.98)	0.96 (0.81–1.14)	0.98 (0.83–1.16)	0.98 (0.73–1.32)	1.00 (0.77–1.29)	0.65 (0.48–0.87)	0.70 (0.54–0.90)
2008/09-2012/13	0.81 (0.73–0.90)	0.82 (0.75–0.90)	1.12 (0.96–1.32)	1.10 (0.93–1.29)	0.81 (0.60–1.09)	0.82 (0.63–1.07)	0.56 (0.42–0.75)	0.61 (0.47–0.79)
2013/14-2017/18	0.90 (0.80-1.00)	0.97 (0.88–1.07)	1.38 (1.16–1.63)	1.43 (1.20–1.70)	0.72 (0.51–1.01)	0.82 (0.60–1.12)	0.55 (0.39–0.77)	0.67 (0.50–0.90)
Test for trend	*p* = 0.01	*p* = 0.11	*p* < 0.0001	*p* < 0.0001	*p* = 0.02	*p* = 0.09	*p* = 0.0001	*p* = 0.001
Socio-economic deprivation quintile†								
1 (least deprived)	1.00	1.00	1.00	1.00	1.00	1.00	1.00	1.00
2	1.23 (1.08–1.41)	1.19 (1.05–1.34)	1.04 (0.86–1.27)	1.01 (0.83–1.23)	1.18 (0.82–1.70)	1.15 (0.82–1.61)	1.34 (0.89–2.02)	1.26 (0.86–1.85)
3	1.20 (1.06–1.37)	1.12 (0.99–1.26)	1.04 (0.86–1.27)	0.95 (0.78–1.16)	0.83 (0.56–1.21)	0.79 (0.55–1.13)	1.36 (0.91–2.04)	1.22 (0.83–1.79)
4	1.38 (1.22–1.57)	1.31 (1.16–1.47)	1.06 (0.88–1.28)	1.07 (0.88–1.30)	1.16 (0.81–1.65)	1.10 (0.79–1.53)	2.36 (1.62–3.43)	2.03 (1.42–2.88)
5 (most deprived)	1.76 (1.56–1.99)	1.32 (1.18–1.47)	1.31 (1.09–1.56)	1.06 (0.88–1.28)	1.67 (1.20–2.33)	1.27 (0.92–1.74)	2.70 (1.88–3.89)	1.88 (1.32–2.66)
Test for trend	*p* < 0.0001	*p* < 0.0001	*p* = 0.002	*p* = 0.38	*p* = 0.001	*p* = 0.15	*p* < 0.0001	*p* < 0.0001

IRR – incidence rate ratio OTC – over-the-counter medicines

*mutually adjusted for sex, age, calendar period, socio-economic deprivation, geographical region

†Socio-economic deprivation quintile – based on index of multiple deprivation (IMD) quintile

‡Refers to ICD-10 categories included within each substance group (further detail supplied in Table 2 and supplementary Table 1)

### Changes over time

Poisonings changed very little over time for 5-11-year-olds, but did show changes over time for 0-4-year-old children, varying according to substance (Fig. 2). When all medicinal poisonings were examined together, after adjusting for sex, age, IMD quintile and geographical region there was no statistically significant change over time (test for trend *p* = 0.11). When examined by substance group however, paracetamol poisonings increased over time (test for trend *p* < 0.0001), DWRD poisonings decreased over time (test for trend *p* = 0.001). There was no significant trend over time for Other OTC poisonings (test for trend *p* = 0.09).

Poisonings involving paracetamol increased by 43% over the study period (aIRR 1.43, 95%CI 1.20–1.70 for the period 2013/14-2017/18 compared to 1998/99-2002/03). These patterns can be observed in Fig. 2, where paracetamol poisonings were primarily observed to increase since 2012. DWRD poisonings decreased by 33% over the study period (aIRR 0.67, 95%CI 0.50–0.90 for the period 2013/14-2017/18 compared to 1998/99-2002/03). Figure 2 shows this reduction to primarily have occurred between 2000 and 2004, then remained stable since then.

There was no change in the rate of medicinal poisonings recorded to be from an unspecified substance over the study period (see supplementary Fig. 1).

### Variations by area-level socioeconomic deprivation

A socio-economic deprivation gradient in poisonings was demonstrated for all medicinal substances combined, as well as for DWRD poisonings (test for trend *p* < 0.0001 for both). For all medicinal poisonings combined, after adjusting for sex, age, calendar period, and geographical region, children living in the most deprived areas had a 30% increase in risk of poisoning compared to children in the least deprived areas (aIRR 1.31, 95%CI 1.16–1.47 for quintile 4 and aIRR 1.32, 95%CI 1.18–1.47 for quintile 5) (Table 3). This risk was primarily seen in the two most deprived quintiles compared to the least deprived, with a lesser increased risk in quintiles 2 and 3. DWRDs showed the largest deprivation gradient with children living in the most deprived areas at 88% to 2 times the risk of poisoning compared to those living in the least deprived areas (aIRR 2.03, 95%CI 1.42–2.88 for quintile 4 and aIRR 1.88, 95%CI 1.32–2.66 for quintile 5). Again a lesser increase risk (22–26%) was seen in quintiles 2 and 3 compared to the least deprived. For paracetamol and Other OTCs, although the unadjusted results did show that children living in the more deprived areas had higher rates of poisoning, after adjusting for other factors, no such deprivation gradient existed.

## Discussion

In children aged 0–11 years old, between 1998 and 2018, poisonings from DWRDs decreased by 33%, while paracetamol poisonings increased by 43%. For all medicinal poisonings combined, no change in incidence rates was demonstrated over the study period. Children living in the most deprived quintile areas were at a 30% increased risk of poisoning compared to children living in the least deprived quintile, for all medicinal poisonings combined. However, this risk was roughly 2-fold for prescribed medication (DWRD) poisoning for children living in the most deprived quintile areas compared to children living in the least deprived quintile, but showed no variation by area-level socioeconomic deprivation for over-the-counter medication poisonings.

Mbeledogu previously showed a decrease of 20% in medicinal poisoning admissions in under-5-year-olds in England from 2000 to 2011 [16]. Our analysis suggests no further reduction in overall medicinal poisoning admissions over time, but when examined by the specific medicines involved, different patterns emerge. Paracetamol poisonings increased over time and poisonings from DWRDs decreased. The authors are not aware of comparable recent data from the UK. One study from the USA has previously shown paracetamol poisonings in children decreasing by 16.7% from 2011 to 2016 [26], while another showed no change in over-the-counter medication poisonings in under-5s from 2001 to 2008 [27]. Two studies using poisoning database data showed increases of up to 200% in opioid poisonings in under-5s in the USA from 1997 to 2012 [27, 28]. While Wang et al., using similar data, showed opioid poisonings in under 6 year olds roughly halved between 2010 and 2018 [29]. 

The decrease in poisoning admissions in children aged under 5 from DWRDs is contrary to the increase in prescriptions of such medications seen over time in the UK. Among adults, prescriptions for weak opioids nearly doubled from 2005 to 2012 [8], while prescriptions of strong opioids increased four- to six-fold from 2000 to 2012 [8, 30]. There was a 28% increase in antidepressant prescribing among 6–18-year-olds from 2003 to 2013 in Wales [31], and a doubling of antidepressant prescriptions among > 14-year-olds in the UK from 1995 to 2011 [7]. In combination with the increase in paracetamol poisonings demonstrated, these findings suggest that messages relating to safe storage of medicines may be leading to positive changes in behaviour with prescribed medication, but that medications deemed to be ‘weaker’ or ‘less dangerous’, such as paracetamol may be stored less safely.

The deprivation gradient we have shown in admissions for childhood poisonings updates existing evidence from the UK. From 1995 to 97, in the Trent region of England, hospital admissions for medicinal poisonings were shown to be 2.5 times higher in the most deprived, compared to least deprived quintile [10]. Admission data from 2000 to 2011 in England showed a weakening of the deprivation gradient in unintentional poisonings over time, from 2.37 times the risk for children living in the most deprived areas compared to the least deprived in 2000, decreasing to 1.54 times the risk in 2011 [16]. 

Furthermore, we examined the risk by medicinal substance. Our finding of no deprivation gradient for over-the-counter medications, alongside a 2-fold increased risk for children living in the most deprived areas for the most common prescribed medications (DWRD), suggests medication availability may be a key factor here. Among adults in the UK, there is evidence of a deprivation gradient in prescribing of opioid [8, 21], gabapentinoid [21] and antidepressant [21] medications, with higher prescribing to people living in areas with higher levels of deprivation for each substance.

With evidence that education and the provision of safety equipment improves safe medicines-storage [32, 33], that providing education to families in disadvantaged areas is cost-effective in promoting safe storage of medicines [34], and that safety equipment schemes reduce childhood injuries [35], the current findings would strengthen the argument that measures such as home safety assessments, as recommended by NICE [36], should be targeted at households in areas of higher deprivation.

To our knowledge, this is the largest and most up to date study examining trends in the specific medicinal substances involved in childhood poisonings in the UK. As CPRD has been shown to be broadly representative of the UK population [37], these results should be generalisable to the wider UK and other similar countries. By using routinely collected healthcare data for both outcomes and exposures, we have minimised the risk of recall or reporting bias.

Our study focussed on the three most common groups of medicinal poisonings, which accounted for 59.7% of poisoning events. The substances involved in the remaining poisoning events were very heterogeneous, with small numbers for specific substances as shown in supplementary Table 1, precluding analysis of poisoning incidence rates by age, sex, deprivation and over time. We did not combine these heterogeneous substances into one group for analysis as the implications for prevention vary by substance (e.g. injectable drugs such as insulin, anaesthetic and therapeutic gases, topical agents, drugs most likely to be prescribed for older adults (e.g. cardiovascular drugs prescribed for grandparents)). Whilst there was some missing data in our study, this is unlikely to have had an important impact on our findings as there were only 4.6% of medicinal poisoning events for which the substance wasn’t specified and 0.9% of events where it was not specified if it was a medicinal or non-medicinal poisoning.

We were not able to include poisonings recorded only in primary care data due to insufficiently detailed recording of poisoning substances. We have therefore not captured less severe poisoning episodes, such as those presenting only to the Emergency Department or the GP, without the need for inpatient admission. Therefore, we cannot assume that our findings are generalisable to poisonings not requiring in-patient admission.

Reporting poisoning intent in this study raised some challenges. Previous evidence shows that 92% of poisoning episodes in 0-4-year-olds and 73% in 5-9-year-olds are unintentional [15]. However, the HES codes used to record outcomes in our study usually did not report intent. Therefore, it was not possible for us to report poisoning intent, although existing evidence suggests these episodes would mostly be unintentional poisonings. It was only possible to obtain deprivation data based on the IMD score of an individual’s GP practice address, which may not always accurately represent residence-based deprivation level. Coding accuracy is a potential source of bias in all database studies as data were primarily recorded for clinical and administrative purposes. However, both CPRD [38] and HES [39] have been shown to be valid for a variety of different disease outcomes.

In studies such as this, the categorisation of medications as ‘over-the-counter’ can sometimes be problematic. For instance, a number of medications, such as paracetamol, can be bought over the counter without a prescription in the UK, but can also be prescribed. The medications in the ‘over-the-counter’ group within our study would most commonly be obtained without a prescription, however in some instances these medications may have been prescribed. This does not alter the key messaging around such medications, which is that they can be dangerous if ingested by children and should be stored safely, in a raised, locked cupboard out of the reach of children, in the same way that prescribed medicines, potentially perceived as ‘more dangerous’ should be.

In summary, poisonings from dependence/withdrawal risk drugs have decreased but those from paracetamol have increased, between 1998 and 2018. The higher poisoning incidence among children living in more deprived areas relate to prescribed, rather than OTC medication. This strengthens the argument that households in more socioeconomically deprived areas will have the greatest need for, and potential to benefit from measures to improve safe storage of medicines and these households are likely to require targeted interventions. It is also important that universal promotion of the safe storage of OTC and prescribed medicines is provided by prescribers, community pharmacies and other outlets of such medication.

### Electronic supplementary material

Below is the link to the electronic supplementary material.

Supplementary Material 1

## Data Availability

This study uses data from the Clinical Practice Research Datalink obtained under licence from the UK Medicines and Healthcare products Regulatory Agency. The data is provided by patients and collected by the NHS as part of their care and support. The interpretation and conclusions contained in this study are those of the author/s alone. HES data Copyright © (2023), re-used with the permission of The Health & Social Care Information Centre. All rights reserved.

## Abbreviations

CPRD: Clinical Practice Research Datalink
HES: Hospital Episode Statistics
GP: General practitioner
ICD-10: International Classification of Diseases, 10th revision
NICE: National Institute for Health and Care Excellence
DWRD: Dependence/withdrawal risk drugs
OTC: Over–the-counter
95%CI: 95% confidence intervals
IRR: Incidence rate ratios
IMD: Index of Multiple Deprivation 2010
aIRR: Adjusted incidence rate ratios
IQR: Inter-quartile range
PY: Person-years
UK: United Kingdom
USA: United States of America
