# Supplementary material for: Patterns and trends of medicinal poisoning substances: a population-based cohort study of injuries in 0–11 year old children from 1998–2018
Source: Arch Public Health. 2024 Apr 16;82:50. doi: 10.1186/s13690-024-01268-7 (PMC11020325; doi:10.1186/s13690-024-01268-7)
Supplement: Supplementary file 1 — Supplementary Material 1 [file 13690_2024_1268_MOESM1_ESM.docx]

Supplementary Table 1. Poisonings by ICD10 category

| **Substance** | **Total no. poisoning events including substance*** | **% of poisoning events including substance (n=3,685)** |
| --- | --- | --- |
| T36 Systemic antibiotics | 57 | 1.5 |
| T37 Other systemic anti-infectives and antiparasitics | 31 | 0.8 |
| T38 Hormones and their synthetic substitutes and antagonists, not elsewhere classified (**excludes** T38.3) | 55 | 1.5 |
| T38.3 Insulin and oral hypoglycaemic [antidiabetic] drugs | 69 | 1.9 |
| T39 Non-opioid analgesics, antipyretics and antirheumatics (**excludes** T39.0, T39.1 & T39.3) | 36 | 1.0 |
| T39.0 Salicylates *(aspirin)* | 55 | 1.5 |
| T39.1 4-Aminophenol derivatives *(paracetamol)* | 1225 | 33.2 |
| T39.3 Other nonsteroidal anti-inflammatory drugs [NSAID] | 198 | 5.4 |
| T40 Opioids (T40.0 Opium, T40.1 Heroin, T40.2 Other opioids, T40.3 Methadone, T40.4 Other synthetic narcotics) | 146 | 4.0 |
| T40.5 Cocaine, T40.6 Other and unspecified narcotics, T40.7 Cannabis (derivatives), T40.8 Lysergide [LSD], T40.9 Other and unspecified psychodysleptics [hallucinogens] | 11 | 0.3 |
| T41 Anaesthetics and therapeutic gases | 11 | 0.3 |
| T42 Antiepileptic, sedative-hypnotic drugs (**excludes** T42.4 & T42.6) | 86 | 2.3 |
| T42.4 Benzodiazepines | 81 | 2.2 |
| T42.6 Other anti-epileptic and sedative hypnotic drugs | 52 | 1.4 |
| T43 Psychotropic drugs, not elsewhere classified (**excludes** T43.0, T43.1, T43.2, T43.6) | 87 | 2.4 |
| T43.0 Tricyclic and tetracyclic antidepressants, | 94 | 2.6 |
| T43.1 Monoamine-oxidase-inhibitor antidepressants, | 1 | <0.1 |
| T43.2 Other and unspecified antidepressants *(includes SSRIs)* | 134 | 3.6 |
| T43.6 Psychostimulants with abuse potential | 70 | 1.9 |
| T44 Drugs primarily affecting the autonomic nervous system (**excludes** T44.7) | 119 | 3.2 |
| T44.7 Beta-adrenoceptor antagonists, not elsewhere classified | 96 | 2.6 |
| T45 Primarily systemic and haematological agents, not elsewhere classified (**excludes** T45.0, T45.2, T45.4) | 72 | 2.0 |
| T45.0 Antiallergic and antiemetic drugs | 105 | 2.8 |
| T45.2 Vitamins, not elsewhere classified | 39 | 1.1 |
| T45.4 Iron and its compounds | 50 | 1.4 |
| T46 Agents primarily affecting the cardiovascular system | 162 | 4.4 |
| T47 Agents primarily affecting the gastrointestinal system | 76 | 2.1 |
| T48 Agents primarily acting on smooth and skeletal muscles and the respiratory system (**excludes** T48.5) | 76 | 2.1 |
| T48.5 Anti-common-cold drugs | 229 | 6.2 |
| T49 Topical agents primarily affecting skin and mucous membrane and ophthalmological, otorhinolaryngological and dental drugs | 229 | 6.2 |
| T49.2 Local astringents and local detergents | 44 | 1.2 |
| T50 Diuretics and other unspecified drugs, medicaments and biological substances | 78 | 2.1 |
| Unspecified medicinal | 169 | 4.6 |

* When added, the total number of events exceeds 3685 because some poisoning events included more than one substance, meaning some events are counted more than once in this table.

Supplementary Figure 1. Unspecified poisoning incidence rate over time, including 95% confidence intervals
